# A Brief Evaluation of Pore Structure Determination for Bioaerogels

**DOI:** 10.3390/gels8070438

**Published:** 2022-07-13

**Authors:** Gabrijela Horvat, Milica Pantić, Željko Knez, Zoran Novak

**Affiliations:** 1 Faculty of Chemistry and Chemical Engineering, University of Maribor, Smetanova 17, 2000 Maribor, Slovenia; gabrijela.horvat@um.si (G.H.); milica.pantic1@um.si (M.P.); zeljko.knez@um.si (Ž.K.); 2 Faculty of Medicine, University of Maribor, Taborska ulica 8, 2000 Maribor, Slovenia

**Keywords:** pore size, pore size distribution, aerogel, gas adsorption, thermoporometry

## Abstract

This review discusses the most commonly employed methods for determining pore size and pore size distribution in bioaerogels. Aerogels are materials with high porosity and large surface areas. Most of their pores are in the range of mesopores, between 2 and 50 nm. They often have smaller or larger pores, which presents a significant challenge in determining the exact mean pore size and pore size distribution in such materials. The precision and actual value of the pore size are of considerable importance since pore size and pore size distribution are among the main properties of aerogels and are often directly connected with the final application of those materials. However, many recently published papers discuss or present pore size as one of the essential achievements despite the misinterpretation or the wrong assignments of pore size determination. This review will help future research and publications evaluate the pore size of aerogels more precisely and discuss it correctly. The study covers methods such as gas adsorption, from which BJH and DFT models are often used, SEM, mercury porosimetry, and thermoporometry. The methods are described, and the results obtained are discussed. The following paper shows that there is still no precise method for determining pore size distribution or mean pore size in aerogels until now. Knowing that, it is expected that this field will evolve in the future.

## 1. Introduction

The term “aerogel” was initially introduced in 1931, describing the jelly material from which the liquid was removed without its structure collapsing [1]. Since the preparation was tedious, limited research was performed in the following years. The new era of aerogels began in the late 1960s and early 1970s with the invention of a faster production of silica aerogels. At the time, most of the research was performed on inorganic aerogels. The broad popularisation of organic aerogels began two decades ago when it was shown that they possess excellent mechanical properties and low thermal conductivity [2]. Moreover, they are biodegradable, biocompatible, and have a large surface area, which may benefit the decomposition of pharmaceuticals [3], metals [3], or other compounds. Organic aerogels are divided into two categories, polymer and carbon aerogels. Bioaerogels belong to the polymer aerogel group and they are most often prepared from polysaccharides or proteins [4]. The latest trends in bioaerogels are focused on the development of new, usually hybrid materials with exceptional properties that could meet the market needs [5]. The definition of aerogel is written in the Gold Book, stating that an aerogel is a “gel, comprised of a microporous solid in which the dispersed phase is a gas”. Undoubtedly, this definition needs some revising since aerogels are much more than microporous solids with gas in their pores. In the literature, aerogels are usually classified as highly porous materials, with porosities of up to 99% and high surface areas obtained after supercritical drying of wet gels.

Aerogel characterization is one of the major topics in the field since the properties of those materials influence their final application. The most important and most often discussed parameters are surface area, porosity, mechanical stability, density, gel structure, thermal conductivity, and pore size distribution (PSD), together with the mean pore size. Specific surface area is determined by the nitrogen adsorption analysis by the BET (Brunauer-Emmett-Teller) method. Bulk density is usually determined by simply measuring sample mass (weighing) and volume (dimensions). The skeletal density of open-pore materials is measured with a helium pycnometer. The most challenging part of aerogels characterization is, without doubt, the determination of their pore size and PSD. This aerogel’s characteristics play a critical role in many applications, including thermal insulation, biomedical applications, etc. Therefore, it is crucial to report it correctly. Simple methods, such as scanning electron microscopy (SEM) offer a 2D view of the surface or cross-section of aerogel. On the other side, it does not offer a full understanding of the 3D structure. Such simple methods are used for the rough estimation of the aerogel’s structure. They could not be used to precisely determine pore sizes, primarily due to the ununiformed structure, tiny pore sizes, and irregularities of pore shapes. It is similar for 3D tomography, from which one cannot obtain PSD below a few hundred nanometres [6].

More precise methods for the determination of PSD and mean pore size exist, but the results are often misleading. This paper will then describe the most employed method for determining those two characteristics and discuss the obtained results to evaluate the method of choice or help critically examine and assess impacts on PSD and mean pore size in future scientific publications.

## 2. Pore Size Determination

**Figure 1:**
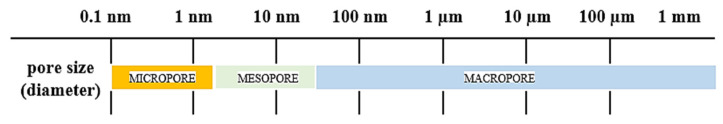
Pore size classification.

In the literature, aerogels are mainly described as mesoporous materials. By the metric system, the word nano means “one billionth or 10^−9^, and the word micro means one millionth” or 10^−6^. However, the IUPAC classification system classifies nanopores into three parts, as presented in Figure 1. Pores with a size below 2 nm are micropores, and this should not be confused with the prefix micro from the metric system. Pores in size between 2 and 50 nm are mesopores, and above 50 nm are macropores. So, all porous materials are classified into one of those three categories depending on their pore size. From this classification, aerogels are mesoporous materials, which means that most of their pores are in the range from 2 nm to 50 nm, as opposed to the IUPAC definition, stating that the aerogels are microporous materials. However, this does not mean that aerogels do not have smaller or larger pores from mesopores, and this is the major challenge with characterizing aerogels for their PSD and mean pore size. Commonly used methods for determining the mean pores size or PSD usually cannot detect broad sizes and the whole pore size range. Often, the micropores and macropores are excluded leading to false results.

**Figure 2:**
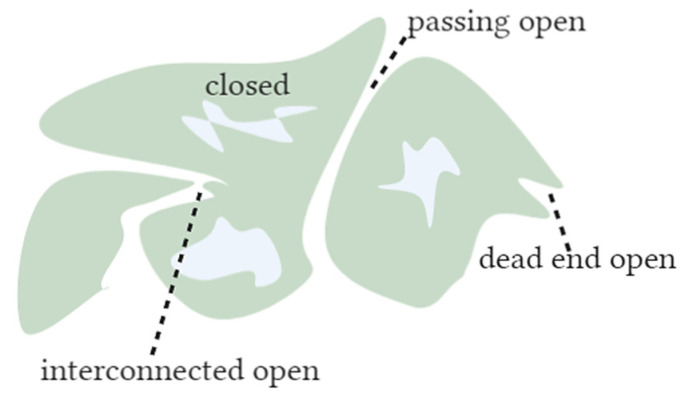
Pore types.

Porous materials are characterized to determine their pore size, pore shape, and accessibility to the surface of the materials. They usually have different pores, from closed, passing open, dead-end open, and interconnected open (Figure 2). As the name already says, a closed pore is completely closed from all sides, and its surface is not accessible. A dead-end open pore is accessible from the surface but does not go to the downstream surface like a passing open pore. Even though this classification does not seem to be highly important for determining the pore size, it is quite the opposite. The pore accessibility and shape significantly affect the mean pore size and PSD in characterization methods such as gas sorption. 

## 3. Gas Sorption

Gas sorption is the characterization method mainly used to determine the surface area of different materials, including aerogels. Surface area is usually determined by the BET method. However, this method is also applicable for determining PSD and mean pore size in aerogels, usually as the first choice technique.

Briefly, this method consists of analyzing a small piece of an aerogel sample in a tube. The adsorption isotherm is obtained, and from those data, different models are applied to determine the desired information, such as the surface area, pore volume, pore size, or PSD. Various gases can be used to analyze samples, including nitrogen and argon, krypton, or CO_2_.

Initially, the sample should be outgassed under a vacuum in order to remove residual moisture, gases, and other impurities. After this step, the sample is prepared for analysis. The system is kept at the boiling point of the gas in between (N_2_ at 77 K, Ar at 87 K) during the analysis. While the system is under vacuum, the sample is cooled to cryogenic temperature. Afterward, the adsorbent gas is introduced to the sample tube. After each dosing, the pressure equilibrium is reached, and then the amount of adsorbed gas is calculated. This step is repeated over a wide range of relative pressures, and adsorption isotherms are obtained as a result. Adsorption isotherm offers valuable information, such as the amount of gas required to form a monolayer (one molecule thick) at the surfaces of the porous sample from which the surface area is calculated. Depending on the type of the adsorption isotherm, the material is classified as micro, meso, or macroporous material [7]. 

**Figure 3:**
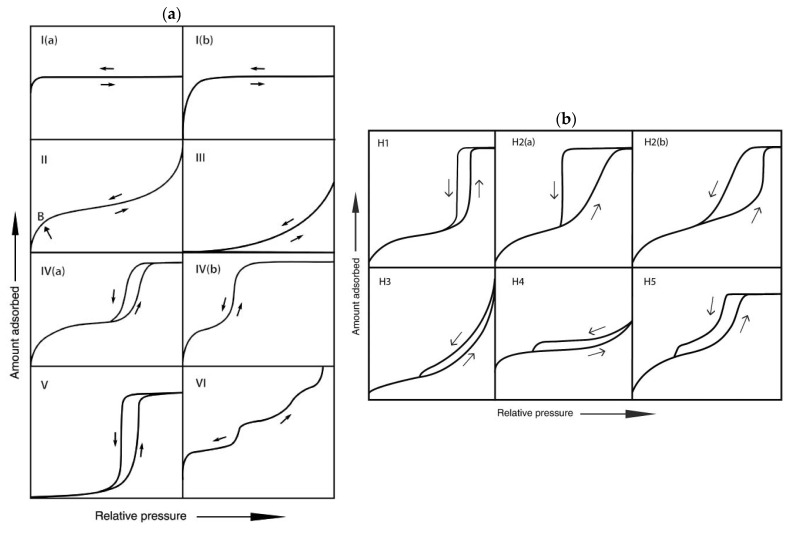
(**a**) The IUPAC Classification of adsorption isotherms for gas–solid equilibria. (**b**) The classification of hysteresis loops (reprinted from [9] with permission, © IUPAC, De Gruyter, 2015).

The 1985 IUPAC classified the adsorption isotherms into six different types [8]. However, some modifications to this standard classification were made in 2015, considering new characteristic types of isotherms (Figure 3a). The type I isotherm is characteristic of microporous materials or materials with tiny pores having relatively small external surface. Type I(a) presents the microporous materials having mainly narrow micropores and type I(b) isotherms presents materials with pore size distribution over a broader range with wider micropores and possibly narrow mesopores. Type II and III are common for nonporous or macroporous materials (materials with wide pores). Type IV(a) and V have a hysteresis loop, which means that the adsorbent molecules have a higher affinity to one another than for the sample, and therefore, they exhibit capillary condensation. Mesoporous materials with smaller width pores are having type IV(b) hysteresis [9]. Type VI is not standard and usually represents completely nonporous materials with a uniform structure [8].

Most of the adsorption isotherms obtained for highly porous aerogels are type IV. Aerogels often have cavities in a variety of shapes and sizes, narrowly and broadly distributed. They may be interconnected and linked to each other, creating an intricate network of pores. The presence of constrictions that regulate desorption from larger interconnected cavities causes the hysteresis loop, characteristic of the type IV isotherm. According to the IUPAC classification, this means that aerogels are primarily mesoporous materials. The shape of this curve provides precious information on the porous structure of the material.

Further classification of adsorption isotherms with a hysteresis loop gives different possibilities; H1, H2(a), H3, and H4 were identified in the original IUPAC classification. H1 is associated with porous materials with narrow pore size distribution and uniform, cylindrical-like pore shape. H2 hysteresis is typical for materials with more complex structures. H2(a)is typical for pore-blocking in a narrow range of pore necks and H2(b) in wider necks [9]. Hysteresis H3 does not have limiting adsorption at higher P/$P_{0}$, which could be the result of non-rigid aggregates of plate-like particles or an assemblage of slit-shaped pores [10]. Such results are not reliable in terms of PSD and mean pore size. Similar to H2, H4 is also associated with complex materials with both micro and mesopores. Characteristic step-down is present in both H3 and H4 isotherms. The H5 type is unusual and is associated with materials that have open and partially blocked mesopores [9].

Further classification of adsorption isotherms for aerogels leads from type IV to type H1. Adsorption isotherm for aerogels is associated with capillary condensation; pores are open-ended cylindrical-shaped mesopores. From the adsorption isotherm, it is possible to obtain the result on the PSD and mean pore size, and there are numerous methods for this. First and foremost is the Barrett-Joyner-Halenda (BJH) method, which operates by the Kelvin Equation (Equation (1)) [11]. However, it is problematic whether to use adsorption or the desorption side of the isotherm for the final determination of PSD in aerogels. Many studies reported pore size or PSD from the branch’s adsorption side; however, the Kelvin equation also uses the formation of a meniscus, which does not exist during adsorption. Groen et al. [7] use the adsorption site because it is hardly affected by the tensile strength effect. However, different physical phenomena can alter the adsorption process, leading to misleading results on micro-and mesopore size.

(1) $$log\frac{P}{P_{0}}=\frac{-c_{K}}{r_{K}}$$

where $c_{K}$ has dimensions of length, and $r_{K}$ is the man radius of curvature of the meniscus inside the pore. 

Another part of the isotherm is its desorption part. PSD obtained from this side of the branch may also be misinterpreted due to the tensile strength effect [7] and the mechanical deformation of aerogels due to capillary condensation [12]. In general, the BJH method also does not include the influence of the solid–fluid interaction on capillary condensations. To summarise, this consequently means that the mean pore size in materials is usually underestimated. Generally speaking, the results for PSD and mean pore size for aerogels obtained from the BJH method are not entirely correct. Compared to some modern techniques such as non-local density functional theory (NLDFT) or electron microscopy determination, it is known that the BJH method renders pore sizes smaller than the real ones by as much as several tens of A°.

While performing the nitrogen adsorption experiment for determining the pore size distribution and even pore volume by the BJH method, it is necessary to define the pore size range where the results are valid. The pore size distribution should be calculated at P/$P_{0}$ > 0.35, considering pores from 1.7 nm to 300 nm. However, it should be communicated and taken into account that pores below 300 nm are only 10–20% of the total pore volume in bioaerogels [2,13]. 

The density functional theory (DFT) coupled with Monte Carlo molecular simulations was first proposed by Seaton et al. [12]. It is used to calculate PSD from adsorption isotherms more precisely. Non-local density functional theory (NLDFT) applies best for materials with highly disordered slit-like micropores and spheres [13]. An essential advantage of the NLDFT method is that it allows us to determine the volume of intrawall pores [14]. The NLDFT model compensates for non-uniformity by not assuming nitrogen gas condenses as a half-sphere meniscus, rather than the Barrett-Joyner-Halenda (BJH) analysis that does consider a half-sphere meniscus. The NLDFT model can calculate the average pore size because mesopores on the aerogel surfaces are non-uniform [15]. However, the dominant peaks from NLDFT typically reported in the literature do not necessarily represent the truly dominant pore size within the system and should not be misinterpreted [16]. In NLDFT, the grand thermodynamic potential of fluid is considered whereas the role of the solid is displayed only through an external potential [17].

**Figure 4:**
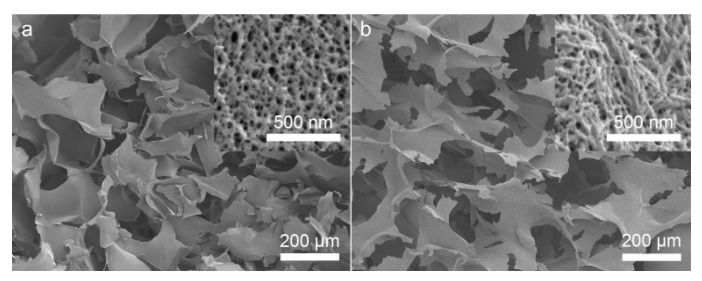
SEM micrographs of cross-sections of (**a**) S-CNC and (**b**) P-CNC aerogels. Both types of aerogels have similar morphology with cross-linked CNC sheets (or flakes) separated by macropores. Insets at higher magnification show similar mesoporous structures of the CNC sheets. Reprinted with permission from [15].

However, if compared to SEM pictures (Figure 4), it is clear that the results from the DFT are again not complete. The mean pore size determined by the gas adsorption was 6 nm and 11 nm for both samples, respectively [15], but the SEM pictures clearly show the presence of macropores larger than 100 µm in diameter. Nitrogen adsorption is not the method for accessing the larger pores, i.e., macropores, and thus, the result obtained by this technique could only be applied to a mesoporous range of the material. Hence, the value for pore size and PSD obtained after the nitrogen adsorption is only related to the mesopores, excluding small micropores and large macropores, and hence it is incomplete. However, it must be mentioned that bioaerogels are also highly prone to the structure deformation during the sample preparation for SEM imaging. Bioaerogels normally have to be gold-sputtered prior SEM imaging due to their non-conductivity. However, it has been proven that gold sputtering greatly affects the structure of aerogels and therefore the results of such analysis should be discussed carefully [18].

Recently, the differential hysteresis scanning (DHS) approach coupling scanning measurements with an advanced modeling framework based on NLDFT has been developed [19]. The DHS technique is a rigorous analysis of the hysteresis loops and subloops observed when scanning an isotherm’s adsorption and desorption branches. Several methods may obtain scanning isotherms. The DHS technique analyses the hysteresis subloops formed by sequential increasing partial saturation of the pore network. It is nowadays considered the first method for quantitatively assessing the architecture of materials with hierarchical pore structures; however, only a few publications reporting this approach have been published until now [19,20]. Nevertheless, this is an excellent tool for describing the range of prevailing structures and their hierarchical interconnections.

Table 1 summarizes the reported pore size of some bioaerogels, as obtained by gas sorption analysis, namely by the BJH and DFT method. As seen from the table, the BJH method is or at least was preferred for determining the pore size in bioaerogels. The DFT method has been used more recently, and there are not many reports on bioaerogels. However, the pore size range obtained by the gas sorption is clearly in the range of mesopores, as expected from the theory. The reported values of gas sorption are, no matter the method used, in the range of 1–50 nm, i.e., the mesoporous range.

Table 2 reports pore sizes of bioaerogels, determined by the SEM.

Another option in using gas sorption analysis is the calculation of the mean pore size ($D_{pore}$) directly from the density of aerogels and their surface area, obtained by the BET method by Equations (2) and (3) [12].

(2) $$V_{pore}=\frac{1}{\rho }-\frac{1}{\rho_{0}}$$

(3) $$D_{pore}=\frac{4V_{pore}}{A}$$

where *ρ* is the bulk density of aerogel and *ρ*_0_ is the skeletal density of aerogel in g/mL. *A* is the specific surface area obtained by gas adsorption with BET method in m^2^/g. Results on the pore size diameter obtained by this calculation were compared to the pore size by the BJH method [69]. Supporting information from the research [69] shows that the pore size, determined by the BJH method, gives values for pore size from 10–16 nm between the samples and the calculated mean pore sizes (Equations (2) and (3)) are between 25–64 nm. However, Equation (3) assumes that the pores have an ideal cylindrical shape and the same volume. Obtained results are usually overestimated since BET method considers only mesopores and small macropores. Lately, only a pore volume obtained from bulk and skeletal densities is reported [70,71] according to Equation (2), most likely to avoid misleading or incomplete results. 

## 4. Mercury Porosimetry

**Figure 5:**
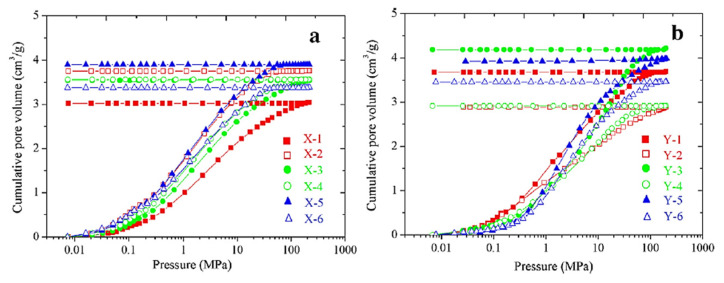
Effect of mercury pressure on specific cumulative pore volume of (**a**) X (80:20 ethanol/H_2_O) and (**b**) Y (50:50 ethanol/H_2_O SiO_2_ aerogels. Reprinted with permission from [74].

Mercury porosimetry is a method for the characterization of the texture of porous materials. Surface area, pore volume, and distributions of pore volume and surface area versus the pore size could be determined by this method. As pressure increases, the mercury penetrates into smaller pores, and the results are then calculated by the Washburn Equation (4) [72]. Mercury porosimetry is the method of choice for the characterization of materials with large macropores [73], for which the intrusion non-wetting mercury occurs at reasonably low pressure. In the case of materials with narrower pores, such as bioaerogels, the intrusion pressure increases strongly, and this can result in the alteration of the pore structure or in the destruction of the material. When a monolithic sample of aerogel is submitted to mercury porosimetry, it is easy to recover and examine the sample after the experiment and to see that its overall volume has strongly decreased and that no traces of mercury are entrapped in the pore network. Although the samples are pressurized during mercury porosimetry, the pore volume varies due to the hierarchical collapse of pores during pressure increases, so the recorded volume variation is practically irreversible during depressurization (Figure 5) [74].

(4) $$d_{p}=-\frac{4\sigma }{p}cos\theta$$

where *p* is the applied pressure in pascals, $d_{p}$ is the pore diameter in meters, *σ* is the surface tension of mercury in newtons per meter, and θ is the contact angle of mercury on the sample in degrees. 

Aerogels commonly contract under pressure applied to mercury, and the Washburn equation cannot be used for building the PSD. Mercury does not enter the pores, and the volume measured is simply the difference between the sample’s initial volume and the volume of the contracting sample [2,12,46,75,76,77]. If in such cases the Washburn equation is used, this leads to pore sizes that are overestimated by several orders of magnitudes.

The relationship between applied pressure (P) and the size (L) of the collapsed pores was first proposed by simple equation Equation (5) [78]:

P = K/L^4^ (5)

The constant K is determined experimentally from linear regression and can vary with the operating conditions of the aerogel synthesis and perhaps the thermal treatment. This equation was later updated by Equation (6) [74]:

(6) $$l=k_{f}/P^{n}$$

where *k_f_* is a constant for buckling, which should be experimentally determined for each material, characterizing the material stiffness; and index *n* expresses the mode of pore destruction under densification pressure. The *k_f_* and *n* are determined from an adjustment of the distribution given by the collapse equation applied to the mercury porosimetry data and the distribution obtained from the nitrogen adsorption isotherm [74]. Mercury intrusion is thus rarely used in bioaerogels. However, quite a few research articles describe this method for characterizing inorganic (silica) aerogels. Figure 5 shows the mercury intrusion curves on silica aerogels. It is shown that the curves are identical between samples. Large volume variation, called pore volume, occurs due to the hierarchical collapse of pores during pressure increases, so the recorded volume variation is practically irreversible during depressurization [74].

Aerogels pressurized under mercury do not accept mercury inside the pores by the intrusion but are compacted [2,77]. Compaction is due to the destruction of pores due to pore edge collapse. Although it is recognized that the pressure of mercury causes the contraction of aerogels, it has sometimes been argued that intrusion occurs at higher pressures so that useful information can be obtained about the distribution of smaller pores. Above transition pressure (P_t_), mercury can enter into the network of small pores, which did not destroy during the collapse step. Therefore, the PSD should be determined by Pirard’s collapse model below P_t_ and by Washburn’s intrusion theory above P_t_ [79]. Pirard’s collapse theory suggests the reconstruction of the macropore size distribution by correlating the equation to the size of the largest pores that have not been compacted after the compression. However, quantitative analysis of the shrinkage that occurs before penetration of the mercury showed that typical aerogels would permit no penetration [80], and only 55–60% volume is taken into account with this method [2].

**Figure 6:**
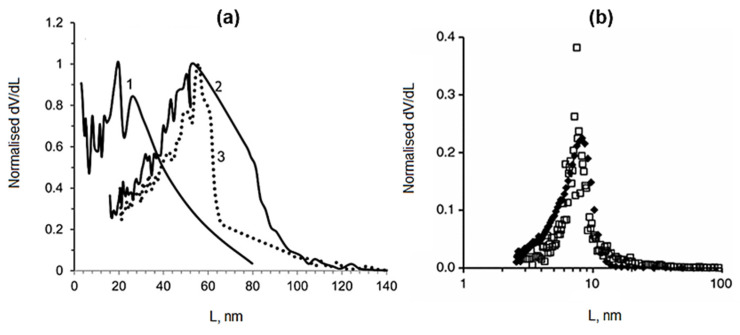
(**a**) Pore size distribution for aeropectins from 3 wt% citrus pectin solution (1, BJH approach; 2, Mercury porosimetry) and from 5 wt% citrus pectin solution (3, Mercury porosimetry). (reprinted from [2] with permission). (**b**) Pore size distribution from thermoporometry (filled square) and from Hg intrusion (open square) for silica gel. (reprinted from [81] with permission).

Figure 6a shows the comparison of BJH gas adsorption method and mercury porosimetry for the determination of the pore size in pectin aerogels. The BJH method gives smaller pore sizes than mercury porosimetry, as expected. PCL-starch aerogels confirm this statement, where in the case of the BJH method, obtained pores ranged between 14 to 15 nm, whereas in the case of mercury intrusion, they were 0.38 to 1.87 µm [59]. Cellulose aerogels showed a great comparison between BJH, mercury intrusion, and the SEM method. Obtained pores for BJH were between 2 and 200 nm, for mercury intrusion between 10 and 250 nm, and for SEM between 5 and 100 nm [47]. However, both methods give significantly different results, as seen from the SEM images, where pores are large, more than few hundred nanometers. This is the result of a really small portion of the total pore volume being evaluated by both methods. In comparison, the mercury intrusion gives similar results to thermoporometry in the case of inorganic silica materials, as reported in Figure 6b.

Table 3 reports pore sizes of bioaerogels, determined by the mercury porosimetry.

## 5. Thermoporometry

Thermoporometry (TPM) is a thermal-related method to measure pore size and PSDs based on the melting point shift of a liquid trapped within a mesoporous medium compared to the surrounding free liquid. The liquid in pores will melt at lower temperatures compared to the bulk liquid. Even though many different liquids have been used for this method, water appears to have several advantages since the large heat of fusion (ΔH_m_ = 334 Jg^−1^) enhances the sensitivity of the differential scanning calorimeter (DSC) technique, which is favorable for the measurement of small-sized samples. However, in some samples, particularly some polymeric aerogels, the use of water is inadvisable because it would cause the material to swell [85]. However, TPM of inorganic silica or even cellulosic aerogels could also be performed in the water due to their usually hydrophobic nature.

**Figure 7:**
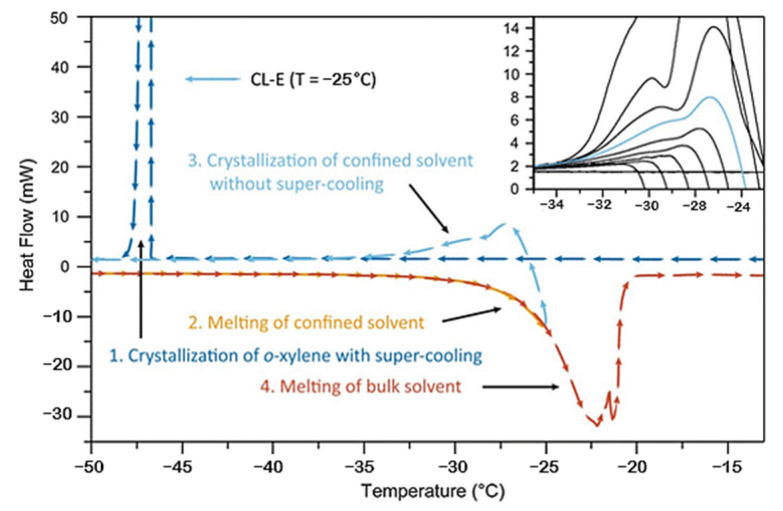
DSC thermogram of aerogels obtained for the determination of pore size by thermoporometry (reprinted from [86] with permission).

Thermoporometry was proposed as an alternative to gas adsorption since the latter covers pore sizes of up to about 170 nm, which is usually insufficient for the broad PSD inherent to most aerogels. Thermoporometry also covers the microporous region and, therefore, usually significantly increases the average pores size in aerogels if compared to nitrogen sorption (BJH) [85]. Thermoporometry is based on the Gibbs-Thomson equation, which quantifies the experimental shift of the melting point of an interstitial liquid caused by its confinement in small pores. The range of applicability of thermoporometry is expected to be much larger than gas sorption since it covers pore sizes beyond 50 nm. Pores up to 430 nm were detected by this method [81]. However, for the organic aerogels, the o-xylene is used as a solvent rather than the water. It was observed that TPM has an approximate size limitation of 200 nm of the radius with water as the probe liquid. This number increases drastically with other probe liquids, for cyclohexane up to 1000 nm [86]. However, the interaction (e.g., swelling) between the material and the liquid must be avoided. Furthermore, when the water is the probe liquid, the slow scanning rate of 0.05 K/min [87] is strongly advised and may increase 2–5 times with organic solvents [86]. The method for TPM is precisely described in [88]. Briefly, 11 thermograms should be obtained for each sample at the ambient atmosphere. First, the sample is cooled to −70 °C, then heated, cooled, and finally heated to 25 °C (Figure 7).

Table 4 collects data about the pore size, obtained by thermoporometry and with BJH method.

The main difference between the two methods is that the gas adsorption method is conducted isothermally as a function of pressure on the dry sample. The TPM is observed isobarically as a function of temperature on the wet sample [89]. As clearly seen from the table, the pore size determination by the BJH method provides clearly underestimated values for pore sizes. Since TPM also detects pores of larger diameters, its applicability in the mesoporous range was proven by the BJH [90]. Comparing both methods, the mesopore size is roughly the same. This is the result of using the Gibs-Thomson constant, which was determined by Schreiber et al. by using pore sizes calculated from gas sorption measurements [87]. Similar results were obtained by Iza et al. [91] for mesoporous silica and poly(N-(2-hydroxyprpyl)methacrylamide). However, it should be taken into consideration that the samples were lyophilised prior to the BJH measurement, and therefore the porous structure may have collapsed during the process, and the lower values of pore size obtained from BJH could partially be attributed to this drying technique, whereas the wet gels were used for the TPM. PSD for porous carbon was also lower in the case of gas adsorption versus TMP [92], but this phenomenon may be common to previous research, i.e., using materials in different states (dry vs. wet). Compared to SEM micrographs, the TPM method shows a good estimation for organic pyrolyzed aerogels [93].

TPM has gained significant interest in recent years, especially for determining the PSD of porous materials. The method has its advantages, such as short analysis time, small sample consumption and affordable experiments. It is also very attractive for analyzing the materials that are prone to collapse during the drying process. However, this does not affect materials that are already in their dry state (e.g., aerogels). In addition, there are other disadvantages. This method is not traditional; its use is not so often as the gas adsorption or mercury porosimetry. The effects of specific interactions between the probe liquid and the material are not completely understood, and this may lead to misinterpretations of DSC signals. The values of the parameters in the Gibbs-Thomson equation are difficult to obtain. These data should be known in advance. In order to avoid this issue, the reference materials with known pore sizes are used in the calibration procedure during the experimental work. Another great concern of this method is the form of crystals within the pores. When the crystal enters a pore, it exerts pressure on the network, and this affects aerogels much the same way as during the mercury porosimetry [94]. The damage is caused by freezing and thawing the samples during this method as the method for determining the pore size distribution of mainly inorganic silica aerogels [95,96] or cellulosic aerogels [88,89,97] at most. Application to other bioaerogels has not been widely reported. 

## 6. Other Characterization Method

Pore size and pore size distribution can be evaluated through other, not yet popularized methods like scattering methods, gaseous thermal conductivity, positron annihilation, or fluid permeation. 

Scattering techniques are quantitative, non-invasive, non-destructive tools for analyzing the PSD of aerogels. Small-angle scattering (SAXS) is sensitive to structures on the scale of 10 µm to few angstroms [98]. The results on the PSD, obtained from SAXS are similar to those obtained by TPM [93]. Many research efforts have employed small angle neutron scattering (SANS) to characterize aerogel structures to examine fractal behaviour at specific length scales and to study changes in structures with respect to processing variables [99]. The drawback of these methods is the fact that the scattering signal does not provide unequivocal information on the structure, and this means that the data evaluation is simple only if one of the well-known characteristics of aerogels is observed.

Positron annihilation lifetime spectroscopy (PAS) is a non-destructive technique used for characterization of single vacancies to mesopores. PAS has extreme sensitivity with respect to small pore size at low densities [100]. The method is found to be suitable for the characterization of mostly microstructures by means of pore-size distribution of inorganic aerogels. Yet the method has not been applied to bioaerogels to the best of our knowledge.

Another approach is NMR cryoporometry, where the basic idea is to detect the shift of the phase transition temperatures for the material that is confined in pores. The shift can be interpreted in terms of pore geometry and can provide information about the pore sizes and pore distribution. The disadvantage in the case of bioaerogels could be the usage of water as the pore-filling liquid which may destroy their sensitive structure [101,102]. 

Classical microscopy methods, such as SEM provide a good visual impression of aerogels. The preparation of the materials for the scanning microscopy usually includes the sputter coating of samples with gold nanoparticles. In addition, soft organic aerogels are often sensitive to the temperature of the scattering beam, and therefore the structure may be destroyed under such conditions. The scanning microscopy of organic aerogels at higher magnifications is hard, and usually, there is no possibility to scan the material at magnifications large enough for the precise characterization of the material. The 2D picture also does not offer a complete insight into the connectivity of the pores, and mostly larger pores are clearly visible. Therefore, this method is useful in combination with some other, before mentioned techniques.

## 7. Conclusions

Pore size distribution and pore size diameter are one of the fundamental physico-chemical properties that should be determined while preparing aerogels for the vast majority of applications. Gas adsorption usually covers the mesoporous range, and mercury porosimetry is used for assessing macropores. Between small macropores and large mesopores, there is a gap, and no single method can provide results with good confidence. The classical BJH model significantly underestimates the mean pore size since it does not include macropores, but it is still widely used in the characterization of porous materials. The DFT model, which has been popularized more recently, seems more appropriate and gives a better estimation of the pore size, but still, it is only applied to the mesoporous range. Differential hysteresis scanning is a recently developed approach for the analysis of hysteresis loops and subloops observed during the gas sorption analysis. Here, the DFT is used for the calculation of the pore size distributions. This method was applied to silica aerogels, but has not been, up to date, utilized in bioaerogels. 

The total pore volume obtained by nitrogen adsorption is less than the actual total pore volumes, calculated by apparent density. This indicates that a considerable portion of the pore volume is not detected by nitrogen adsorption. The undetected volume could be attributed to macropores which could be determined by the mercury porosimetry. However, even with this method applied, the total pore volume is less than the realistic total pore volume; this accordingly suggests that the fraction of pores is not detected, and that could be attributed to micropores. Quantitative analyses indicate that neither of the above-mentioned methods provides an accurate measure of the pore size or pore volume of an aerogel. If those methods are used, they should be applied together, so the broad spectrum of pores is accessed, and then all the possible sources of error should be accounted for and discussed together with the obtained results. Thermoporometry, on the other side, is employed in order to prevent the above-mentioned drawbacks of commonly used methods. However, TPM still requires data for the final calculations in advance. Similarly, like gas adsorption and mercury porosimetry also, thermoporometry can cause compression of the aerogel, which leads to the underestimation of the pore volume in pore size. In addition, there is still not much research reporting the use of this method on bioaerogels, excluding cellulose aerogels. The main reason could lie in the use of water as a solvent for the analysis, which is clearly not a good choice for water-soluble polysaccharides. 

Other methods used for evaluation of pore size and pore size distribution are small-angle scattering, small angle neutron scattering, positron annihilation lifetime spectroscopy, NMR cryoporometry, and classical microscopy methods.

The clear need for a new or optimized technique that could determine the pore size of aerogels in a broad range is highly desired. Since aerogels are highly sensitive to external pressure and their nanostructure may deform during nitrogen adsorption, mercury porosimetry, and even thermoporometry, the new method should take into account this limitation. One option would be the development of a new characterization technique using supercritical technology. 

## Figures and Tables

**Table 1:** Reported pore size or pore size distribution of some bioaerogels, obtained by gas sorption analysis.

Bioaerogel	Method	Pore Size (nm)	Specific Surface Area, m^2^/g	Reference
Agar	DFT	35	320	[21]
		36	320	[22]
Alginate	BJH	11.1–13.1	261–437	[23]
		11.7	150–300	[24]
		14	147	[25]
		10.5–10.9	400–430	[26]
		25.5	125.9	[27]
		10–15	394–590	[28]
		14–28	376–575	[29]
		8.8–20	402–419	[30]
		30.5	359.5	[31]
Alginic acid	BJH	25.1	320	[32]
	DFT	40	375	[21]
	DFT	20	390	[22]
Ca-alginate	BJH	38	570	[33]
	DFT	28	495	[21]
	DFT	37	570	[22]
Cu-alginate	DFT	30	680	[21]
Alginate/pectin	BJH	10.4–14.2	299–417	[23]
Barely beta-glucan	BJH	2.7–2.8	160–167	[34]
Carrageenan	BJH	7.4–16.5	34–174	[35]
	BJH	1.2–2.7	128.8–385.5	[36]
	BJH	19	200	[33]
	DFT	22	230	[21]
	DFT	18	200	[22]
Cellulose	BJH	7.9–34	154–434	[37]
		1–106	55.2	[38]
		7.9–39.4	260–485	[39]
		19	356	[40]
		40–90	147–246	[41]
		7.1–11.7	72–115	[42]
		8.9–11.7	55–310	[43]
		10.5–28.9	2.0–80.7	[44]
		8	250	[45]
		17–30	140–250	[46]
		2–200	/	[47]
Cellulose nanocrystal	DFT	6–11	130–190	[15]
		3.9–11.1	190–320	[48]
Chitosan Chitosan (α-chitin) Chitosan (β-chitin)	BJH BJH BJH BJH BJH DFT DFT DFT DFT	11	330	[33]
		12.6–15	257–479	[49]
		50–120	973	[50]
		21.3–43.6	737–872	[51]
		3.29–11.13	66–845	[52]
		11	330	[21]
		12	330	[22]
		22	210	[21]
		25	150	[22]
		18	560	[21]
β-Chitin	DFT	20	560	[22]
Guar	BJH	15	111	[25]
Pectin	BJH	7.2–26.3	143–593	[53] [23] [2] [54] [55] [56] [23] [25]
		13.1–16.8	272–407	
		11	230–270	
		7.3–28.8	174–485	
		17–22	247–284	
		15.6–17.1	354–386, 272–437	
		11.1–13.117, 19	510, 384	
Pectin/xanthan	BJH	6.8	175–289	[57]
Starch	BJH	9.4	217	[31]
Potato starch		7.2	72.5	[24]
Eurylon7 starch		1.9	90.3	[24]
Wheat starch		19–26	20.2–59.7	[58]
PCL-starch		14.1–15.1	1.3–1.7	[59]
Xanthan	BJH	20	363	[25]
Silk fibroin	BJH	18–24	336–432	[60]
		11–17	260–308	[61]
Whey protein	BJH	12.3–27.4	310–447	[62]

**Table 2:** Reported pore size or pore size distribution of some bioaerogels, obtained by scanning electron microscopy.

Bioaerogel	Method	Pore Size (nm)	Reference
Alginate	SEM	200	[63]
Alginic acid	SEM	11.4 *	[64]
Ca-alginate	SEM	5.8 *	[64]
Ba-alginate		7.4 *	
Co-alginate		6.6 *	
Cu-alginate		4.4 *	
Ni-alginate		6 *	
Cellulose Cellulose nanocrystal	SEM	50–1000	[45]
		5–100	[47]
		125–250	[65]
		50–200	[66]
		7 um	[48]
Chitosan	SEM	>10	[67]
		10–50	[68]

* Fibril size.

**Table 3:** Reported pore size or macropore size distribution of some bioaerogels, obtained by mercury porosimetry.

Bioaerogel	Pore Size (µm)	Reference
Alginate—pectin	0.183–1.081	[82]
Citrus pectin	0.019–0.046	[2]
Cellulose	0.002–0.050	[46]
	0.012–0.025	[46]
	0.7–47.5	[83]
	0.01–25	[47]
Chitosan	56.8–57.04	[84]
PCL-starch aerogel	0.38–1.87	[59]

**Table 4:** Average pore size (Ø) according to thermoporometry analysis and nitrogen sorption experiments at 77 K, TBAF/DMSO (CL-TBAF), [EMIm] [Oac]/DMSO (CL-EMIm), NMMO·H_2_O (CL-NMMO), and Ca(SCN)_2_·8H_2_O/LiCl (CL-CTO) [88].

Method	CL-TBAF	CL-EMIm	CL-NMMO	CL-CTO
Pore size TPM (nm)	36/101	62	96	84
Modal pore size BJH (nm)	34.5	34.4	32.1	2.7
